# zDHHC3-mediated S-palmitoylation of SLC9A2 regulates apoptosis in kidney clear cell carcinoma

**DOI:** 10.1007/s00432-024-05737-y

**Published:** 2024-04-15

**Authors:** Xiuyun Zhang, Junpeng Hou, Guangyuan Zhou, Haixia Wang, Zeang Wu

**Affiliations:** 1 Department of Pathology, Renmin Hospital of Wuhan University, Wuhan, 430000 China; 2 Department of Orthopedic Surgery, Zhengzhou Central Hospital Affiliated With Zhengzhou University, Zhengzhou, 450000 China; 3 School of Basic Medicine, Tongji Medical College, Huazhong University of Science and Technology, Wuhan, 430000 China; 4 School of Medicine, Shihezi University, Shihezi, 832003 Xinjiang Province China; 5 First Affiliated Hospital of Shihezi University, Dr. Zeang Wu, 107 North Second Road, Shihezi, 832003 Xinjiang Province China

**Keywords:** S-palmitoylation, zDHHC3, SLC9A2, Apoptosis, Kidney renal clear cell carcinoma

## Abstract

**Purpose:**

Kidney clear cell carcinoma (KIRC) has a poor prognosis, high morbidity and mortality rates, and high invasion and metastasis rate, and effective therapeutic targets are lacking. zDHHC3 has been implicated in various cancers, but its specific role in KIRC remains unclear.

**Methods:**

In this study, we performed a pan-cancer analysis, bioinformatics analysis, and cell experiment to detect the role of zDHHC3 in KIRC.

**Results:**

zDHHC3 was significantly down-regulated in KIRC, and that its high expression was associated with favorable patient outcomes. We identified 202 hub genes that were most relevant to high zDHHC3 expression and KIRC, and found that they were involved mainly in ion transport and renal cell carcinoma. Among these hub genes, SLC9A2 was identified as a downstream gene of zDHHC3. zDHHC3 suppression led to decreased expression and S-palmitoylation of SLC9A2, which further inhibited the apoptosis of Caki-2 cells.

**Conclusion:**

Our findings suggest that zDHHC3 plays an important role in KIRC, due partly to its regulation of SLC9A2 S-palmitoylation. The targeting of the zDHHC3–SLC9A2 axis may provide a new option for the clinical treatment of KIRC.

**Supplementary Information:**

The online version contains supplementary material available at 10.1007/s00432-024-05737-y.

## Introduction

Renal cell carcinoma (RCC) is the most common type of kidney cancer, accounting for approximately 90% of all cases. Worldwide, an estimated 431,300 cases of kidney cancer were newly diagnosed and 179,400 deaths from kidney cancer occurred in 2020 (Siegel et al. 2021). Kidney clear cell carcinoma (KIRC) is the most critical histological subtype of RCC, accounting for about 80–90% of cases (Biles et al. 2020). The prognosis of KIRC is poor, and the disease shows widespread resistance to chemotherapy and radiotherapy (Zhao et al. 2018). The 3-year KIRC survival rate is less than 5% (Kuehn et al. 2014). Early surgery is currently the primary treatment, but tumor metastasis occurs postoperatively is up to 30% of cases (Hsieh et al. 2017). The efficacy of currently available targeted drugs is suboptimal, highlighting the need to identify biomarkers with diagnostic and prognostic value and to develop sensitive tumor treatment targets.

Protein is the main executor of gene to perform physiological functions, and a series of post-translational modifications (PTM) can regulate the function of protein. PTM can modulate protein biological functions by altering their physical and chemical properties, conformation, and binding capacity (Balakrishnan et al. 2020). Approximately 50–90% of human proteins, including various tumor proteins and tumor suppressors, undergo PTM (Ray et al. 2015). For instance, STAT6 acetylation can inhibit the migration and invasion of KIRC cells and contribute to the survival of KIRC patients (Shao et al. 2023). S-palmitoylation, a reversible form of PTM, can modify protein secretion, transport, and interaction, and membrane trafficking and stability (Wu et al. 2021a, b). Its potential role in cancer treatment has garnered increasing attention, FAK S-palmitoylation mediated by zDHHC5 plays a key role in glioma proliferation, invasion and epithelial mesenchymal transformation (Wang et al. 2024).

zDHHC3, one of 23 human palmitoyl acyltransferases, regulates the S-palmitoylation of various proteins. Several studies have highlighted its critical role in cancer treatment. zDHHC3 knockout accelerated the degradation of laminin-binding integrin α6β4 in various cancer cells, affecting cancer progression, metastasis, and angiogenesis (Sharma et al. 2012). zDHHC3 was found to be significantly upregulated in breast cancer, and mediated protein S-palmitoylation promoted the growth of breast tumors by regulating cell oxidative stress and aging (Sharma et al. 2017). zDHHC3 is also known to modulate the S-palmitoylation of programmed cell death ligand 1 (PD-L1), and the blocking of PD-L1 S-palmitoylation increased the sensitivity of tumor cells to T-cell cytotoxicity (Yao et al. 2019). In contrast, one study found that zDHHC3 was significantly down-regulated in KIRC, and that decreased zDHHC3 expression was associated with poor KIRC prognosis (Liu et al. 2020). The difference in the effect of high zDHHC3 levels in KIRC and other cancers may be due to differences in palmitoylated substrate proteins. The mechanism by which zDHHC3 affects KIRC by regulating the S-palmitoylation of substrate proteins remains unknown.

In this study, we analyzed zDHHC3 mRNA levels in KIRC using the Cancer Genome Atlas (TCGA) and GSE152938 data. Additionally, we examined the effects of zDHHC3 expression on the survival of patients with KIRC. We conducted a gene set enrichment analysis (GSEA) and a weighted gene co-expression network analysis (WGCNA) of zDHHC3-related genes. Finally, we predicted and identified the target genes of zDHHC3 and detected the role of zDHHC3-mediated S-palmitoylation in KIRC. Our findings suggest that zDHHC3’s mediation of S-palmitoylation plays an important role in KIRC, and that the targeting of zDHHC3-mediated protein S-palmitoylation could contribute to the clinical treatment of KIRC.

## Materials and methods

### Data source and screening

We used Sangerbox software to download mRNA data of patients with KIRC from the TCGA database (http://www.sangerbox.com/tool). The Limma R package was used to screen for genes that were differentially expressed between KIRC and healthy samples at the significance level of *P* < 0.05.

### Pan-cancer zDHHC3 gene expression and survival analyses

We used the online tool TIMER 2.0 (Li et al. 2020) to analyze differentially expressed genes (DEGs) in various cancers using TCGA data (https://genemania.org/). We also applied GEPIA (Tang et al. 2017), which uses TCGA and CTEx mRNA sequencing data, to examine differential gene expression between tumor and healthy tissues, and to conduct patient survival and gene expression correlation analyses (http://gepia.cancer-pku.cn/index.html).

### Confirmation of zDHHC3 expression in GSE152938 data

We selected two KIRC samples and one healthy kidney tissue sample from the GSE152938 dataset to characterize zDHHC3 expression at the single-cell level. The Seurat R package was used to implement standard single-cell sequencing data processing pipelines. Filtering was performed to identify cells with < 200 and > 5000 genes and a mitochondrial gene percentage > 10%. Then, the screening was performed to identify DEGs between KIRC and healthy samples using the FindMarkers function.

### GSEA of zDHHC3-related genes

GSEA is a powerful tool for the analysis of genome-wide expression profiles using chip data. Unlike differential analysis, GSEA does not require manual screening for the identification of DEGs, which may result in the missing of critical information. It can be used to identify gene sets that are not very different from one another, but have consistent difference trends. We performed a GSEA of zDHHC3-related genes using the clusterProfiler package, with h.all.v7.3.symbols.gmt serving as the reference gene set. *P* values < 0.05 were considered statistically significant.

### WGCNA of zDHHC3-related genes

WGCNA (Langfelder et al. 2008) is a valuable tool for the evaluation of pairwise correlations between gene expression profiles, the clustering of genes with synergistic changes into modules, and the exploration of associations between gene modules and disease phenotypes. Its first steps are the detection of sample and gene quality, the cluster analysis of samples based on the gene expression, and the deletion of outliers. Then, a similarity matrix is constructed by calculating correlations between all gene pairs and the soft threshold power of β, and determining whether a scale-free network can be established. This matrix is converted into a topological overlap matrix, a dissimilarity matrix is constructed, and co-expressed gene modules are identified by dynamic tree cutting. Finally, highly similar modules are merged based on module-level correlation, and correlations between each module and the disease phenotype are calculated. The hub genes in the most relevant disease module are then screened.

### Gene ontology and Kyoto encyclopedia of genes and genomes analyses

The Database for Annotation, Visualization and Integrated Discovery (Sherman et al. 2021) is an online tool for the analysis of gene and protein functions (DAVID, https://david.ncifcrf.gov/). The main biological function categories analyzed are cell compartment (CC) and molecular function (MF), as well as biological processes (BP). Metascape (Zhou et al. 2019) is another online analytical tool that integrates multiple databases for the examination of biological processes, signal pathways, and protein–protein and protein–drug interactions. *P* value < 0.05 was considered statistically significant (https://metascape.org/gp/index.html).

### Prediction of target genes of zDHHC3

GENEMANIA (Franz et al. 2018) is a website containing data on 166,691 genes and 660,443,499 protein interactions (https://genemania.org/). It can be used to verify various aspects of protein interactions, including co-localization, co-expression, physical interactions, prediction, shared protein domains, and genetic interactions. The data on zDHHC3 and the genes related most closely to it were uploaded to GENEMANIA for the construction of protein–protein interaction (PPI) networks and identification of potential target genes of zDHHC3.

### Confirmation of target gene expression with GSE213324 data

We selected 60 healthy kidney tissues and 63 RCC tissues from the GSE213324 dataset to characterize the expression of zDHHC3 target genes. The Limma R package was used to screen DEGs between the KIRC and healthy samples using the significance threshold of *p* < 0.05.

### Coimmunoprecipitation

Caki-2 and RCC23 cells were lysed with binding buffer containing a protein inhibitor cocktail (1:100), and the protein concentration was determined after centrifugation at 14,000×*g* at 4 ℃ for 10 min. Protein samples (500 μg) were mixed with 3 μg mouse zDHHC3 monoclonal antibody (sc-377378; Santa Cruz) overnight at 4 °C. Then, the antibody–protein complex was mixed with 50 μl protein A/G magnetic beads for 2 h, and washed three times with washing buffer to separate unbound antibodies and proteins. Binding and protein loading buffers (1:4) were then added and the samples were heated at 95 ℃ for 5 min before analysis by western blotting.

### Short hairpin RNA design and transfection

The short hairpin RNA (shRNA) of SLC9A2 and zDHHC3 were synthesized by WZBIO (Wuhan, China). The sequences of SLC9A2 and zDHHC3 were as follows, SLC9A2: 5’-CGCCCATTCTTTGAGAACATT-3’, zDHHC3:5’-**CCCAAAGGAAATGCCACTAAA-3’**, and non-targeted control shRNA served as the negative control (NC). Cells were cultured in Dulbecco’s modified Eagle medium in a 6-cm cell culture dish, and 10 ul shRNA was added at 60–80% confluence. The mixture was incubated at 37 ℃ for 48 h, and the cells were harvested for further analysis.

### Kidney tissue collection

Kidney tissues were collected from seven healthy individuals and seven patients with first-diagnosis KIRC aged 18–70 years at Remnin Hospital of Wuhan University between December 2022 and February 2023. KIRC diagnoses were confirmed by clinical and imaging examinations and histopathological analysis of biopsy or surgical samples. The patients did not receive radiotherapy, chemotherapy, or other anti-tumor treatment before admission. Patients with incomplete clinical data were excluded.

### Immunofluorescence

Paraffin sections of kidney tissues were roasted at 65 ℃ for 45 min to remove wax, and then antigen repair was performed. Sealed with 1% bovine serum albumin solution at room temperature for 1 h, sections were incubated with antibodies (zDHHC3,sc-377378; SLC9A2, 46,256–1; 1:100 dilution) at 4 ℃ overnight and then incubated with fluorescent-labeled secondary antibodies for 1 h. Finally, the nucleus was stained with DAPI for 10 min. The sections were sealed with an anti-fluorescent quencher and stored at 4 °C.

### Acyl–biotin exchange assay

Briefly, protein was enriched by antibody and protein A/G magnetic beads (B23202; Bimake), then incubated with 50 mM N-ethylmaleimide (N80860; Macklin) at room temperature for 4 h. Samples were divided into two equal parts and added 1 M Hydroxylamine (H828371; Macklin) and NaCl, respectively. Next, the samples were incubated with Biotin-HPDP (5 μM, A8008; Apexbio) at room temperature for 1 h, then added 1 × loading buffer (without mercaptoethanol) and heated at 95 ℃ for 5 min, and subsequent western blot analysis was performed.

### Western blot

The concentration of protein extracted from cells was determined by BCA assay kit, then sodium dodecyl sulfate–polyacrylamide gel electrophoresis (SDS-PAGE) was used to separate proteins. The proteins were electrotransferred to polyvinylidene difluoride (PVDF) membranes and then blocked with 5% skim milk at room temperature for 1 h. Next, membranes were incubated with primary antibody at 4℃ overnight, and the secondary antibody was incubated at room temperature for 1 h. The blots were visualized and analyzed with chemiluminescent reagents and ImageJ software. The following primary antibodies were used: zDHHC3 (sc-377378, 1:500; Santa), P21-activated kinase 7 (PAK7; 35,292–1, 1:500; SAB), solute carrier family 9 member A2 (SLC9A2, also sodium–hydrogen exchanger 2; 46,256–1, 1:500; SAB), caspase 3 (19,677–1-AP, 1:500; Proteintech), and β-actin (66,009–1-LG, 1:10,000; Proteintech).

### Statistical analysis

SPSS 22.0 software was used for analysis and graphpad prism 8.0 software was used for plotting. The data in box plots are presented as means ± standard deviations and were analyzed using the unpaired Tukey test. All experiments were repeated successfully five times and yielded consistent results. The significance level was set to *p* < 0.05.

## Results

### Pan-cancer zDHHC3 expression

**Fig. 1:**
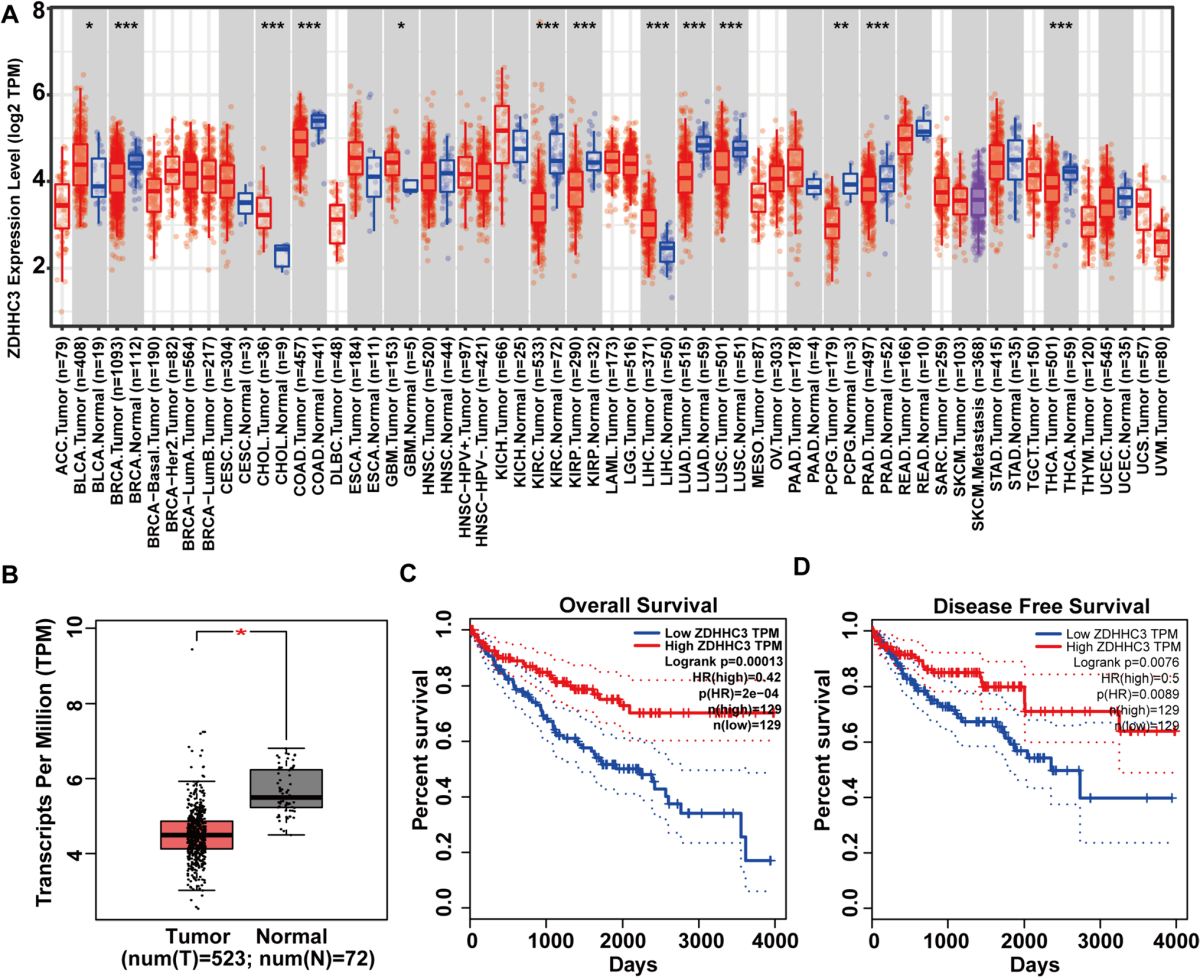
Pan-cancer zDHHC3 expression and association with survival. **A** zDHHC3 expression in 32 human cancers relative to healthy tissues. TCGA data, Wilcoxon test. **B** zDHHC3 expression in paired KIRC and healthy tissues. CEPIA data, one-way analysis of variance. **C**, **D** Overall and disease-free survival of patients with KIRC according to zDHHC3 expression. Groups were defined by quartiles. Log-rank test. *p < 0.05, **p < 0.01, ***p < 0.001

We analyzed the expression of zDHHC3 in 32 human cancers to explore its potential roles in cancer-suppressing or carcinogenic effects. The TIMER data indicated that zDHHC3 was significantly upregulated in BLCA, CHOL, GBM, and LIHC and down-regulated in BRCA, COAD, KIRC, KIRP, LUAD, LUSC, PCPG, PRAD, and THCA relative to its expression in adjacent healthy tissues (Fig. 1A). Consistently, GEPIA data showed that zDHHC3 was remarkably down-regulated in KIRC (Fig. 1B).

### Prognostic value of zDHHC3 for KIRC

We next performed survival analysis to determine the diagnostic value of zDHHC3 for KIRC. We set the cutoff for high (vs. low) zDHHC3 expression at 50%. We analyzed overall survival (OS) and disease-free survival (DFS). In patients with KIRC, high zDHHC3 expression levels had favorable effects on OS and DFS (Fig. 1C, D). In addition, we observed a significant negative correlation between zDHHC3 expression and the KIRC stage (Supplementary Fig. 1A).

### zDHHC3 was down-regulated in KIRC samples at the single-cell level

**Fig. 2:**
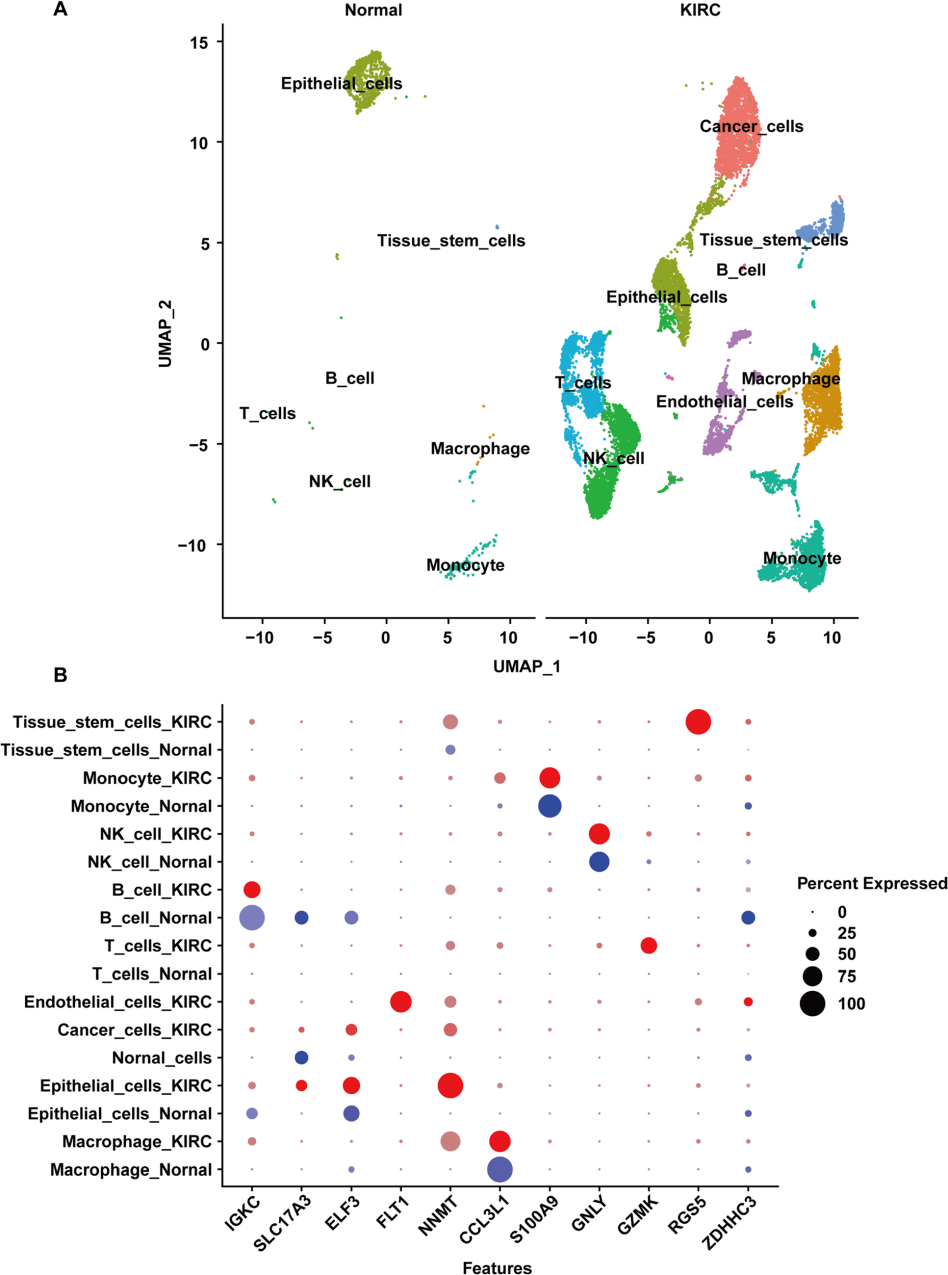
zDHHC3 expression in KRIC at the single-cell level. **A** UMAP plot showing cell types (represented by colors) of control and KIRC samples. **B** Expression patterns of cell markers and zDHHC3 in various cell types

To confirm the down-regulation of zDHHC3 expression in KIRC, we analyzed KIRC single-cell sequencing data. After quality control, we obtained 12,916 cells from two KIRC samples and 925 cells from one control kidney tissue sample. The top 2000 highly variable genes in single cells were selected for principal component analysis. Uniform manifold approximation and projection (UMAP) analysis was performed. The UMAP analysis led to the identification of 24 cell clusters, and we screened for marker genes in each cell cluster. We used the single R R package to annotate cell types and identified a total of eight cell types (Fig. 2A). We identified 4918 DEGs and found that zDHHC3 was significantly down-regulated in cancer cells relative to healthy cells (Fig. 2B).

### GSEA of zDHHC3-related genes

**Fig. 3:**
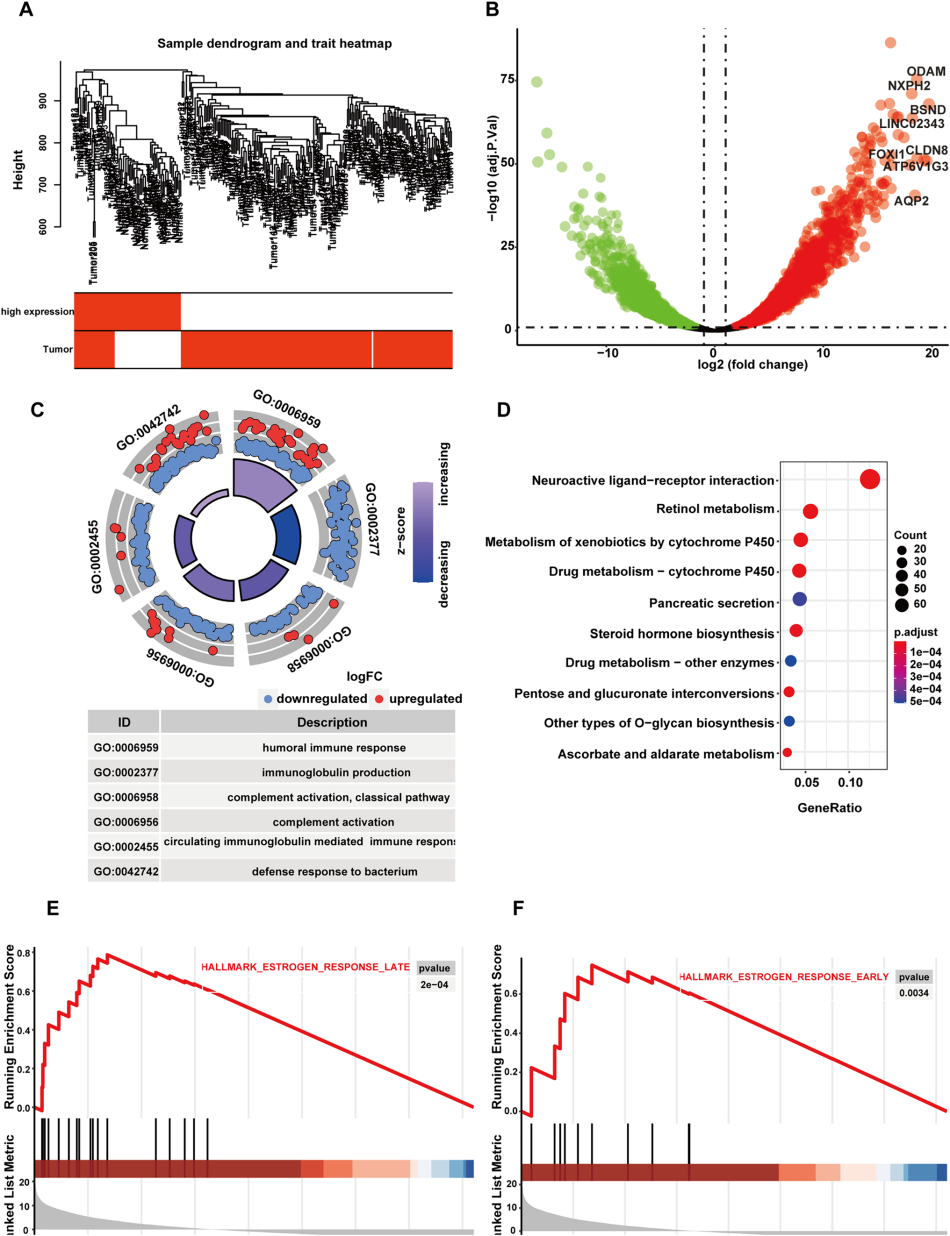
GO, KEGG, and GSEA findings for zDHHC3-related genes. **A** Sample dendrogram and trait heatmap. **B** Volcano plot of all gene expression changes in high vs. low zDHHC3 expression samples. Red and green represent upregulated and down-regulated genes, respectively, and named genes have logFC values > 18. **C** Circle plot of GO terms enriched in zDHHC3-related genes. (**D**) Bubble chart of pathways enriched in zDHHC3-related genes. **E**, **F** GSEA-derived plots of late and early estrogen response enrichment

To further investigate the changes in signal pathways resulting from zDHHC3 expression in KIRC, we conducted gene ontology (GO) and Kyoto Encyclopedia of Genes and Genomes (KEGG) analyses after regrouping the KIRC samples. We ranked zDHHC3 expression in healthy and KIRC samples from lowest to highest, and defined the bottom and top 25% of samples as the low and high expression groups, respectively (Supplementary Fig. 1B). After removing the outliers, we included 251 (206 tumor and 45 healthy, 180 low and 71 high zDHHC3 expression) samples in the subsequent analysis (Fig. 3A, Supplementary Table 1). We used the Limma R package to identify DEGs between the low and high expression groups. In total, we identified 5040 DEGs (2122 upregulated and 2918 down-regulated) with adjusted *p* values < 0.05 and |log_2_^fold change (FC)^ |> 1. DEGs with log2 FC values > 18 are shown in Fig. 3B. The GO analysis revealed that the immune response was the most enriched biological process in the high zDHHC3 expression group (Fig. 3C).

The KEGG analysis showed that genes in the high zDHHC3 expression group were involved mainly in neuroactive ligand–receptor interaction and many metabolic processes, including retinol, xenobiotic, drug, ascorbate, and aldarate metabolism (Fig. 3D). The GSEA showed that nine gene sets were significantly up-regulated in the high zDHHC3 expression group (Table 1). These gene sets were involved mainly in the estrogen response, Kras signaling, bile acid metabolism, myogenesis, and xenobiotic metabolism. The enrichment of the top two significantly upregulated gene sets is shown in Fig. 3E and F.

**Table 1:** GSEA results of zDHHC3-related genes

Description	Enrichmentscore	NES	*p* value	*p* adjust	*q* values
Hallmark_estrogen_response_late	0.7871	2.2312	0.0002	0.0013	0.0005
Hallmark_estrogen_response_early	0.748	1.8319	0.0034	0.0171	0.0072
Hallmark_bile_acid_metabolism	0.6532	1.6449	0.0215	0.0403	0.0169
Hallmark_apical_junction	0.6449	1.624	0.0252	0.042	0.0177
Hallmark_kras_signaling_up	0.6287	1.7257	0.0114	0.0316	0.0133
Hallmark_spermatogenesis	0.5862	1.6818	0.0126	0.0316	0.0133
Hallmark_myogenesis	0.5786	1.7454	0.0067	0.0251	0.0106
Hallmark_kras_signaling_dn	0.5378	1.9906	0.0001	0.0013	0.0005
Hallmark_xenobiotic_metabolism	0.5227	1.6133	0.0196	0.0403	0.0169

### WGCNA of zDHHC3-related genes

**Fig. 4:**
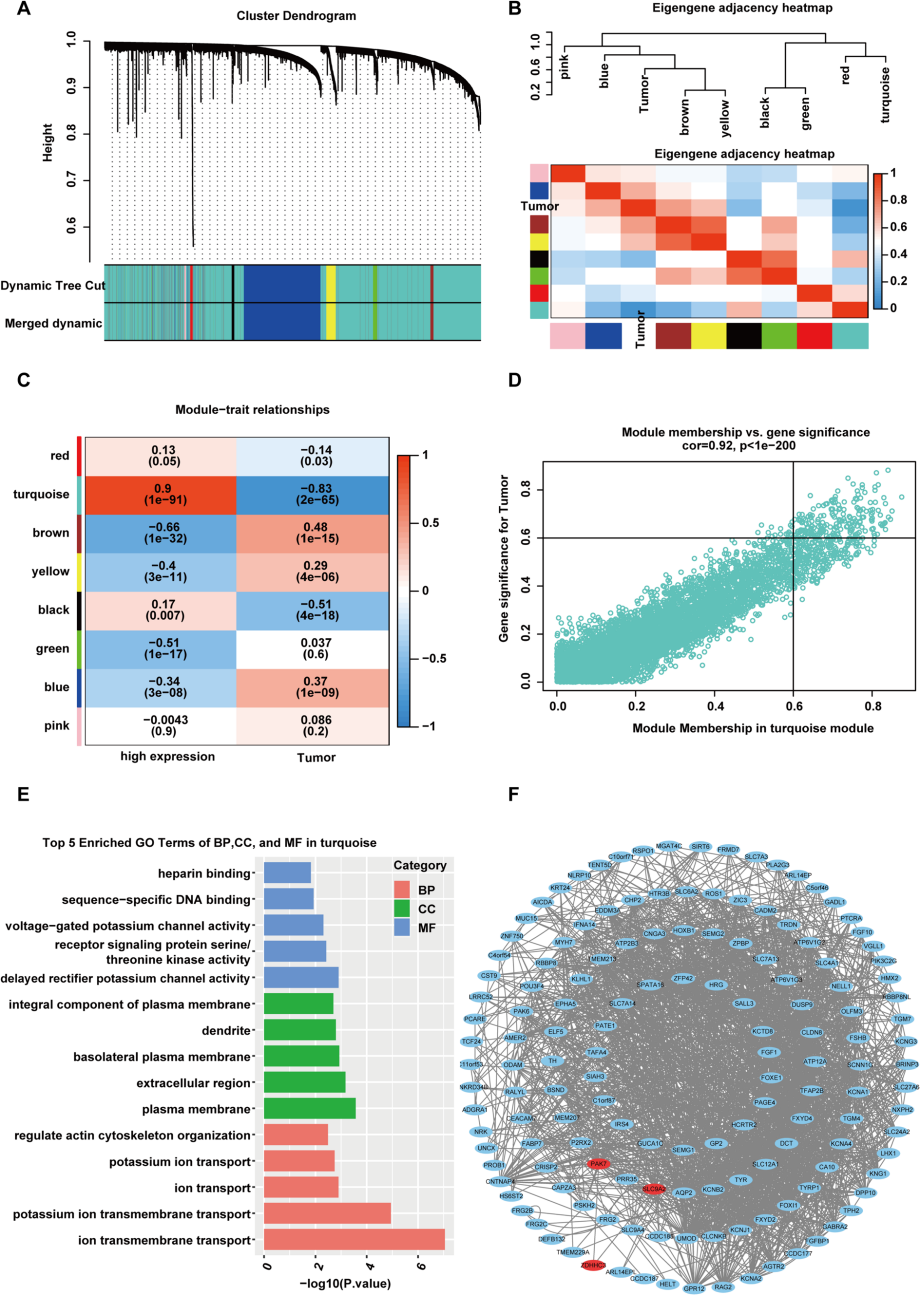
WGCNA findings for zDHHC3-related genes. **A** Cluster dendrogram of all zDHHC3-related genes used to detect co-expression modules (represented by colors). **B** Eigengene adjacency heatmap of all modules and disease phenotype. **C** Correlations of modules with clinical traits. **D** Correlation between MM and GS in the turquoise module. **E** Top five enriched GO terms of BP, CC, and MF in the turquoise module. **F** PPI network of hub genes in the turquoise module

To assess the gene changes related to high zDHHC3 expression and their impacts on KIRC occurrence and development, and to identify zDHHC3-related oncogenes and potential therapeutic targets, we conducted a WGCNA of the top 25% of genes (*n* = 8210) in the 251 samples. The soft threshold power of β was set to 3 (scale-free *R*^2^ = 0.894) to establish a scale-free gene network (scale-free *R*^2^ = 0.87; Supplementary Fig. 1C, D). We identified modules with > 30 genes from the network and merged modules with dissimilarity values < 0.2. Eight modules were identified in the network (Fig. 4A). Genes not included in these modules were excluded from subsequent analysis.We then constructed a network heatmap to detect interaction between modules and their associations with KIRC. All modules were divided into two clusters associated positively (cluster 1; Fig. 4B, pink, blue, brown, and yellow modules) and negatively (cluster 2; Fig. 4B, black, green, red, and turquoise modules) with KIRC. Among the eight modules, the turquoise module was associated positively with high zDHHC3 expression and negatively with KIRC (Fig. 4C). We calculated gene significance (GS) and module membership (MM) values for each gene in the turquoise module using intramodular analysis; genes with GS and MM values > 0.6 were considered to be essentially related to KIRC (Fig. 4D). We uploaded 202 genes obtained in the previous step to DAVID to perform the GO analysis. The most enriched GO terms for BP, CC, and MF were ion transmembrane transport, plasma membrane, and delayed rectifier potassium channel actively, respectively (Fig. 4E). The KEGG analysis revealed that the kidney cell carcinoma and taste transduction pathways were significantly enriched in the high zDHHC3 expression group.

### SLC9A2 interacts and co-localizes with zDHHC3

**Fig. 5:**
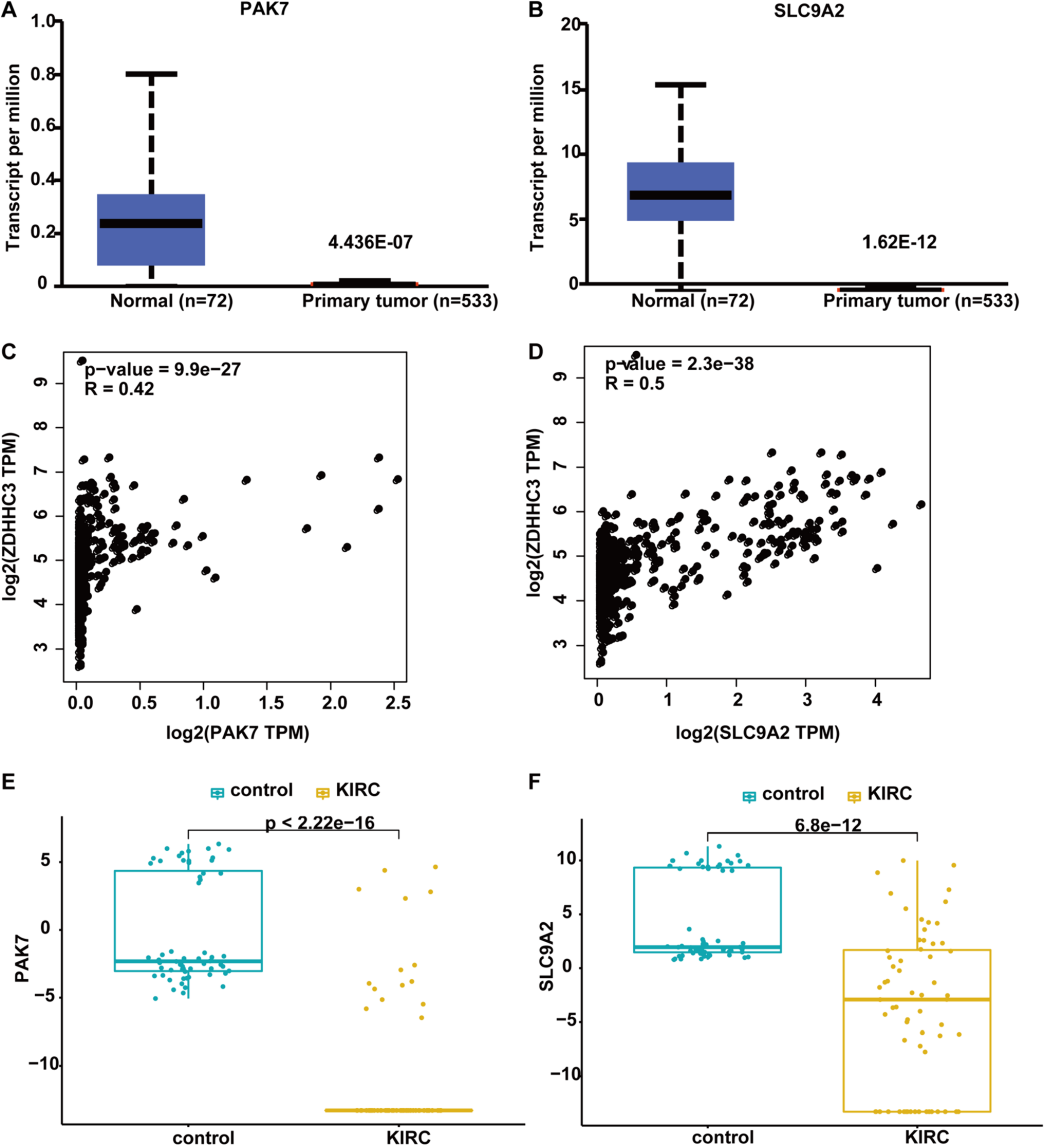
PAK7 and SLC9A2 expression in the CEPIA and GSE213324 datasets. **A**, **B**, **E**, **F**) PAK7 and SLC9A2 expression in tumor and healthy tissues. CEPIA (**A**, **B**) and GSE213324 (**E**, **F**) data, Wilcoxon test. **C**, **D** Correlations of PAK7 and SLC9A2 expression with zDHHC3 expression

**Fig. 6:**
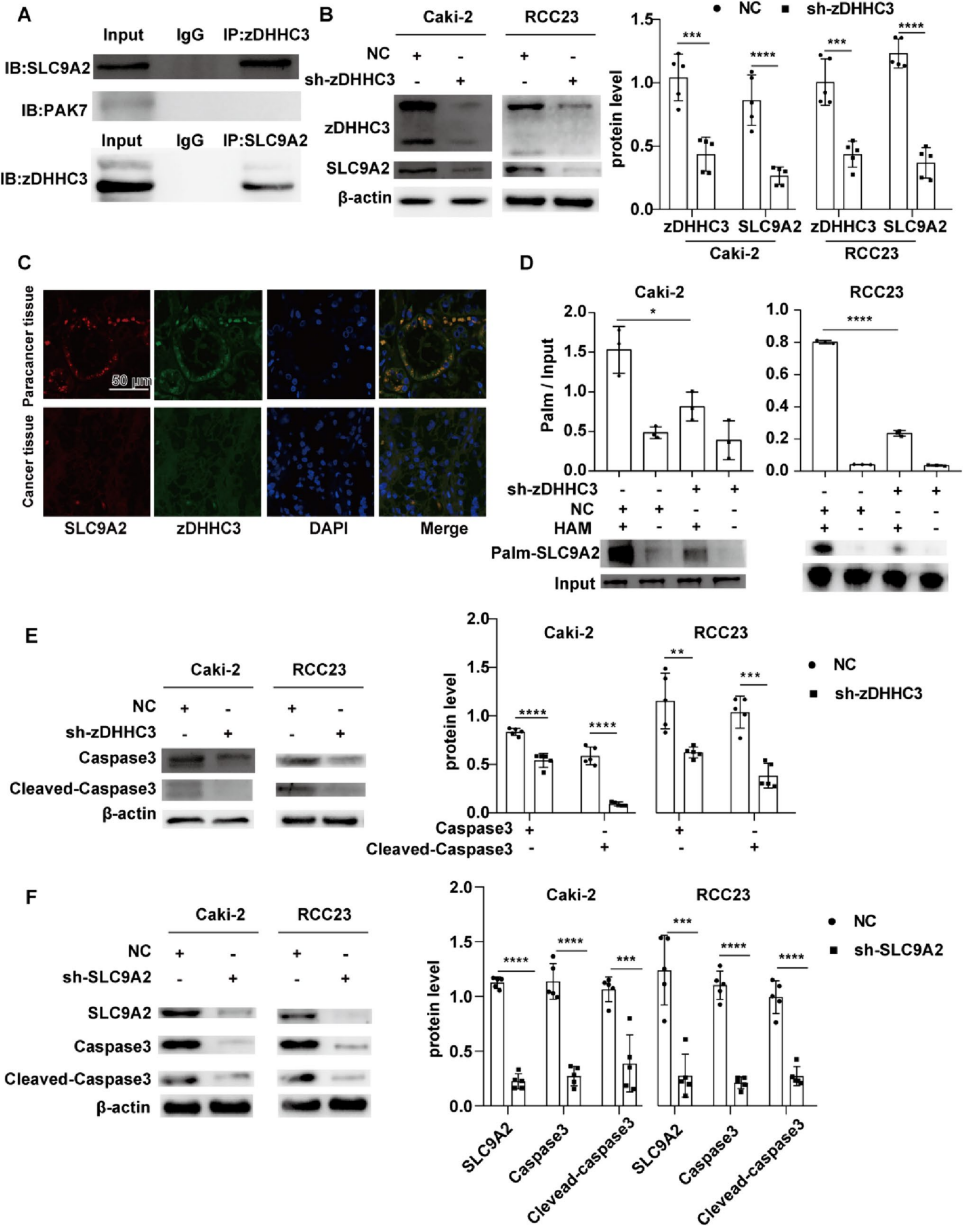
SLC9A2 is the downstream gene of zDHHC3. **A** Co-IP images of SLC9A2 interaction with zDHHC3 in Caki-2 cells. **B** zDHHC3 knockdown decreased the SLC9A2 protein level in Caki-2 and RCC23 cells. **C** Immunofluorescence images of zDHHC3 and SLC9A2 in KIRC and healthy kidney tissues. **D** The effect of zDHHC3 knockdown on the S-palmitoylation of SLC9A2, the ratio of “Palm” to “Input” represents the S-palmitoylation level of SLC9A2. **E** The effect of zDHHC3 knockdown on the apoptosis level of Caki-2 and RCC23 cells. **F** SLC9A2 knockdown decreased the apoptosis level of Caki-2 and RCC23 cells. *p < 0.05, ***p < 0.001, ****p < 0.0001

We used GENEMANIA to construct a PPI network by uploading these genes along with zDHHC3, and found that PAK7 and SLC9A2 were predicted to interact with zDHHC3 (Fig. 4F). Subsequently, we analyzed their expression in KIRC and observed that they were significantly down-regulated in KIRC relative to their expression healthy samples (Fig. 5A, B). We also employed the CEPIA database to analyze the correlation of correlation of zDHHC3 with PAK7 and SLC9A2 expression, and found that the expression of both genes correlated positively with zDHHC3 expression (Fig. 5C, D). Moreover, the expression of PAK7 and SLC9A2 was significantly down-regulated in KIRC samples from the GSE213324 dataset (Fig. 5E, F). To further confirm these relationships, we performed in-vitro experiments and found that zDHHC3 interacted only with SLC9A2. zDHHC3 knockdown with shRNA significantly reduced the protein level of SLC9A2, and immunofluorescence analysis of healthy and KIRC kidney tissues revealed the co-localization of SLC9A2 and zDHHC3 (Fig. 6A–C). These results suggest that SLC9A2 is the downstream gene of zDHHC3.

### zDHHC3 regulates the S-palmitoylation of SLC9A2

As zDHHC3 could regulate protein S-palmitoylation, We performed an acyl–biotinyl exchange assay to detect whether zDHHC3 konockdown regulated the S-palmitoylation level of SLC9A2, and found that zDHHC3 inhibition significantly suppressed the S-palmitoylation of SLC9A2, suggesting that zDHHC3 was responsible for regulating SLC9A2 S-palmitoylation (Fig. 6D). As some studies have shown that zDHHC3 plays a key role in cell apoptosis, we detected the apoptosis level of cells in the NC and shRNA groups. As expected, zDHHC3 suppression significantly reduced the apoptosis level of Caki-2 and RCC23 cells (Fig. 6E). Moreover, SLC9A2 knockdown showed similar inhibitory effect on the apoptosis level of Caki-2 and RCC23 cells, suggesting that zDHHC3 could influence cell apoptosis by regulating the S-palmitoylation of SLC9A2(Fig. 6F).

## Dscussion

S-palmitoylation modification is essential for the maintenance of normal tumor-promoting and tumor-suppressing protein functions. Previous findings suggest that the targeting of melanocortin-1 receptor S-palmitoylation could prevent melanomagenesis (Chen et al. 2017, 2019). Thus, an understanding of how protein S-palmitoylation affects the functions of individual proteins is of utmost importance. As a regulatory enzyme for protein S-palmitoylation, zDHHC3 has been shown to play a critical role in the initiation and progression of many cancers. However, our understanding of the role of zDHHC3 in KIRC is limited, and further research is necessary. This study demonstrated that zDHHC3 is significantly down-regulated in KIRC and that high zDHHC3 expression is a protective factor in KIRC patients. Samples with high zDHHC3 expression were significantly enriched in genes and pathways associated with immune response–related biological processes, suggesting that zDHHC3 plays a role in immune-related diseases by regulating the S-palmitoylation of some proteins associated with immune responses. zDHHC3 deletion was found to reduce the S-palmitoylation level of ACE2, impairing its membrane localization and secretion into extracellular vesicles, which play key roles in COVID-19 (Xie et al. 2021). In addition, the zDHHC3-mediated S-palmitoylation of interferon-induced transmembrane protein 3 was found to be essential for the restriction of numerous viral infections (Mcmichael et al. 2017).

In this study, the hub genes from the module identified as most relevant to KIRC and high zDHHC3 expression were involved mainly in ion transport and kidney cell carcinoma. The ion transport mechanism has been found to be a critical driver of cancer (Litan et al. 2015) and to occur in multiple stages of cancer, including the transition from healthy to cancer cells (Hanahan et al. 2022). The Ca^2+^, K^+^, and Cl^−^ channels have been reported to play essential roles in the regulation of cell proliferation and cancer development (Ko et al. 2013). For instance, Ca^2+^ signaling is crucial for the regulation of cancer cell proliferation, migration, and invasion, and for cell death (Iamshanova et al. 2017). The K^+^ and Cl^−^ channels affect cell migration by regulating the swelling and contraction of different cell areas (Michelucci et al. 2023). Thus, the changes in the ion transport caused by high zDHHC3 expression play an essential role in cancer.

As an ion transport protein,SLC9A2 is responsible for the exchange of intracellular H^+^ for external Na^+^ for participation in Na^+^ transport and the regulation of cell pH and volume (Muthusamy et al. 2013). SLC9A2 was identified as the downstream gene of zDHHC3 in this study, and SLC9A2 was co-located and interacted with zDHHC3, which contribute to the S-palmitoylation of SLC9A2 catalyzed by zDHHC3. Enhanced S-palmitoylation might facilitate the membrane localization of SLC9A2, further promote its physiological function. Moreover, Cys-112 was predicted as the palmitoylation site of SLC9A2 by CSS Plam 4.0 software, which needs to be verified by further cell experiment. The cell volume is related closely to the cell cycle (Rivarola et al. 2017; Zuccolini et al. 2023), and SLC9A2 inhibition significantly suppressed cell apoptosis in our study. In addition, zDHHC3 regulated the S-palmitoylation of SLC9A, and protein S-palmitoylation has been related to apoptosis (Frohlich et al. 2014). Taken together, this evidence suggests that zDHHC3 regulates the apoptosis of KIRC cells by inhibiting SLC9A2 S-palmitoylation and expression.

## Conclusions

We found that zDHHC3 was significantly down-regulated in patients with KIRC, and higher zDHHC3 levels were associated with a better prognosis. The high zDHHC3 expression group showed enrichment in genes related to the immune response and ion transport. zDHHC3 could inhibit SLC9A2 expression and S-palmitoylation, thereby suppressing KIRC cell apoptosis.

### Supplementary Information

Below is the link to the electronic supplementary material.

Supplementary file1 (TIF 293 KB)

Supplementary file2 (DOCX 23 KB)

## Data Availability

The data generated in the present study may be requested from the corresponding author.
