# Supplementary figures and images for: zDHHC3-mediated S-palmitoylation of SLC9A2 regulates apoptosis in kidney clear cell carcinoma
Source: J Cancer Res Clin Oncol. 2024 Apr 15;150(4):194. doi: 10.1007/s00432-024-05737-y (PMC11018659; doi:10.1007/s00432-024-05737-y)

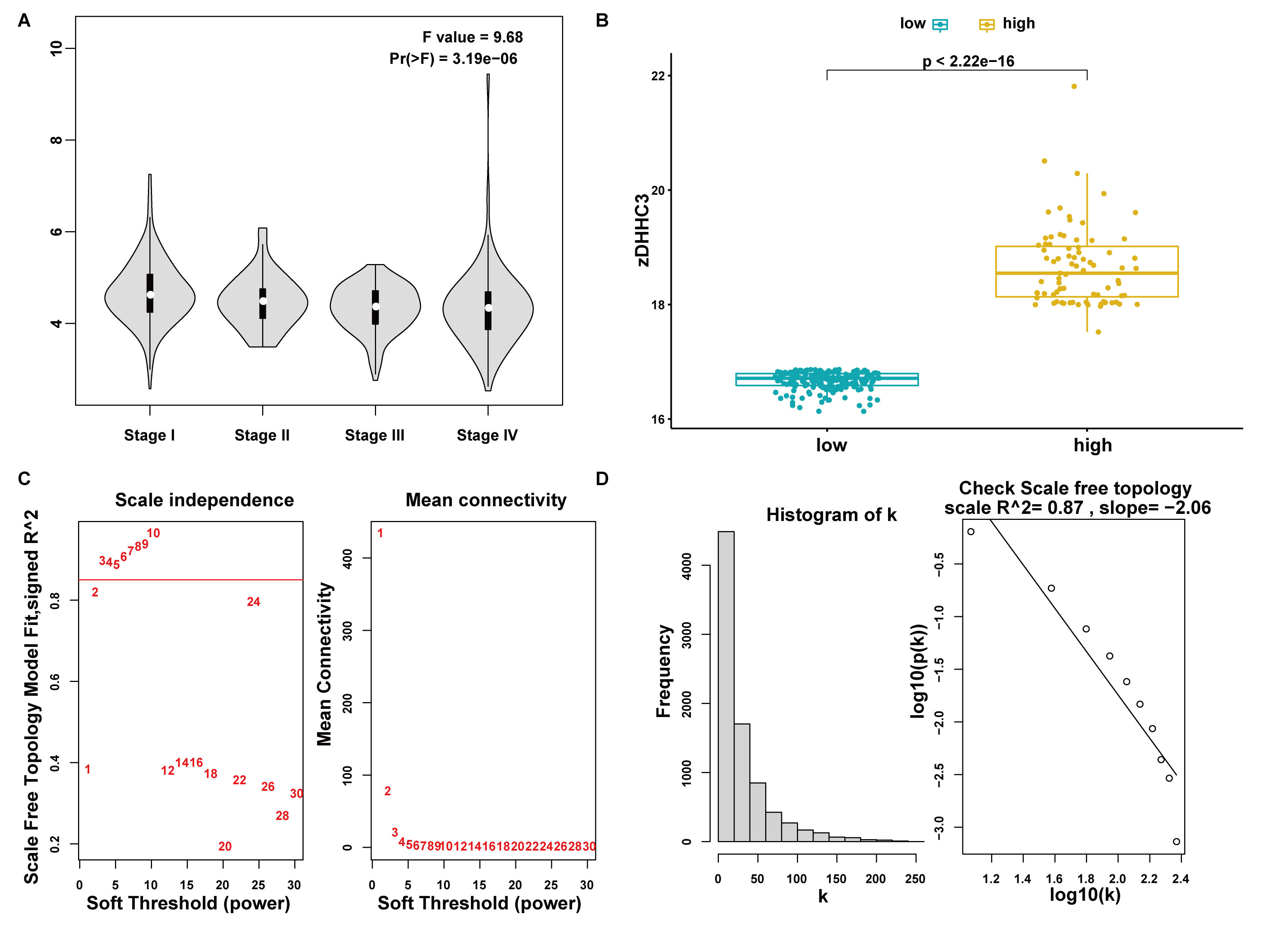

Supplement: Supplementary file 1 — Supplementary file1 (TIF 293 KB) [file 432_2024_5737_MOESM1_ESM.tif]
