# Supplementary material for: zDHHC3-mediated S-palmitoylation of SLC9A2 regulates apoptosis in kidney clear cell carcinoma
Source: J Cancer Res Clin Oncol. 2024 Apr 15;150(4):194. doi: 10.1007/s00432-024-05737-y (PMC11018659; doi:10.1007/s00432-024-05737-y)
Supplement: Supplementary file 2 — Supplementary file2 (DOCX 23 KB) [file 432_2024_5737_MOESM2_ESM.docx]

1. **Table 1** The basic information of TCGA samples in zDHHC3 high/low expression group.

| TCGA ID | zDHHC3 | Tumor | TCGA ID | zDHHC3 | Tumor | TCGA ID | zDHHC3 | Tumor |
| --- | --- | --- | --- | --- | --- | --- | --- | --- |
| TCGA.B4.5832.01 | low | 1 | TCGA.B8.4143.01 | low | 1 | TCGA.B0.5099.01 | low | 1 |
| TCGA.B4.5843.01 | low | 1 | TCGA.B0.5102.01 | low | 1 | TCGA.B0.5705.01 | low | 1 |
| TCGA.B8.4143.01 | low | 1 | TCGA.B0.5104.01 | low | 1 | TCGA.B0.5703.01 | low | 1 |
| TCGA.B8.4146.01 | low | 1 | TCGA.B0.5094.01 | low | 1 | TCGA.B0.5711.01 | low | 1 |
| TCGA.B8.4153.01 | low | 1 | TCGA.B0.5113.01 | low | 1 | TCGA.B0.5698.01 | low | 1 |
| TCGA.B8.4154.01 | low | 1 | TCGA.B0.5115.01 | low | 1 | TCGA.B0.5699.01 | low | 1 |
| TCGA.B8.4622.01 | low | 1 | TCGA.B0.5116.01 | low | 1 | TCGA.MM.A564.01 | low | 1 |
| TCGA.B8.5158.01 | low | 1 | TCGA.B0.5399.01 | low | 1 | TCGA.EU.5907.01 | low | 1 |
| TCGA.B8.5159.01 | low | 1 | TCGA.B0.5402.01 | low | 1 | TCGA.CJ.4916.01 | low | 1 |
| TCGA.B8.A54D.01 | low | 1 | TCGA.B0.5692.01 | low | 1 | TCGA.G6.A8L7.01 | low | 1 |
| TCGA.B8.A54F.01 | low | 1 | TCGA.B0.5693.01 | low | 1 | TCGA.G6.A8L8.01 | low | 1 |
| TCGA.B4.5843.01 | low | 1 | TCGA.B0.5696.01 | low | 1 | TCGA.DV.A4W0.01 | low | 1 |
| TCGA.B8.A54I.01 | low | 1 | TCGA.CZ.5989.11 | high | 0 | TCGA.EU.5904.01 | low | 1 |
| TCGA.BP.4161.01 | low | 1 | TCGA.DV.5576.01 | high | 1 | TCGA.DV.A4VX.01 | low | 1 |
| TCGA.BP.4164.01 | low | 1 | TCGA.A3.3328.01 | high | 1 | TCGA.CZ.5987.01 | low | 1 |
| TCGA.A3.3387.01 | low | 1 | TCGA.A3.3374.01 | high | 1 | TCGA.CZ.5986.01 | low | 1 |
| TCGA.BP.4165.01 | low | 1 | TCGA.AK.3427.01 | high | 1 | TCGA.CZ.5989.01 | low | 1 |
| TCGA.BP.4174.01 | low | 1 | TCGA.AK.3447.01 | high | 1 | TCGA.CZ.5470.01 | low | 1 |
| TCGA.BP.4176.01 | low | 1 | TCGA.AK.3433.01 | high | 1 | TCGA.CZ.5984.01 | low | 1 |
| TCGA.BP.4325.01 | low | 1 | TCGA.AK.3440.01 | high | 1 | TCGA.CZ.5465.01 | low | 1 |
| TCGA.BP.4326.01 | low | 1 | TCGA.BP.4761.01 | low | 1 | TCGA.CZ.5463.01 | low | 1 |
| TCGA.BP.4327.01 | low | 1 | TCGA.AS.3777.01 | high | 1 | TCGA.B0.4833.01 | low | 1 |
| TCGA.BP.4335.01 | low | 1 | TCGA.AK.3465.01 | high | 1 | TCGA.CZ.5462.01 | low | 1 |
| TCGA.BP.4340.01 | low | 1 | TCGA.B0.4712.11 | high | 0 | TCGA.CZ.5459.01 | low | 1 |
| TCGA.BP.4346.01 | low | 1 | TCGA.B0.4699.01 | high | 1 | TCGA.CZ.5460.01 | low | 1 |
| TCGA.BP.4351.01 | low | 1 | TCGA.B0.4700.11 | high | 0 | TCGA.CZ.4864.01 | low | 1 |
| TCGA.BP.4761.01 | low | 1 | TCGA.B0.5117.01 | high | 1 | TCGA.CZ.5453.01 | low | 1 |
| TCGA.BP.4768.01 | low | 1 | TCGA.B0.4834.01 | high | 1 | TCGA.CZ.5454.01 | low | 1 |
| TCGA.BP.4775.01 | low | 1 | TCGA.B0.5083.01 | high | 1 | TCGA.CZ.4857.01 | low | 1 |
| TCGA.BP.4165.01 | low | 1 | TCGA.B0.5694.11 | high | 0 | TCGA.CZ.4858.01 | low | 1 |
| TCGA.BP.4964.01 | low | 1 | TCGA.B0.5690.11 | high | 0 | TCGA.CZ.4859.01 | low | 1 |
| TCGA.BP.4969.01 | low | 1 | TCGA.B0.5697.11 | high | 0 | TCGA.A3.3306.01 | low | 1 |
| TCGA.BP.4973.01 | low | 1 | TCGA.B0.5701.11 | high | 0 | TCGA.A3.3319.01 | low | 1 |
| TCGA.BP.4981.01 | low | 1 | TCGA.B0.5703.11 | high | 0 | TCGA.A3.3320.01 | low | 1 |
| TCGA.BP.4983.01 | low | 1 | TCGA.B0.5705.11 | high | 0 | TCGA.A3.3322.01 | low | 1 |
| TCGA.BP.4985.01 | low | 1 | TCGA.B0.5711.11 | high | 0 | TCGA.A3.3324.01 | low | 1 |
| TCGA.BP.4989.01 | low | 1 | TCGA.B2.3923.01 | high | 1 | TCGA.A3.3335.01 | low | 1 |
| TCGA.BP.5000.01 | low | 1 | TCGA.CZ.5982.11 | high | 0 | TCGA.A3.3343.01 | low | 1 |
| TCGA.BP.5001.01 | low | 1 | TCGA.CZ.5984.11 | high | 0 | TCGA.A3.3346.01 | low | 1 |
| TCGA.BP.5004.01 | low | 1 | TCGA.CZ.5470.11 | high | 0 | TCGA.A3.3351.01 | low | 1 |
| TCGA ID | zDHHC3 | Tumor | TCGA ID | zDHHC3 | Tumor | TCGA ID | zDHHC3 | Tumor |
| TCGA.BP.5007.01 | low | 1 | TCGA.CZ.5467.11 | high | 0 | TCGA.A3.3352.01 | low | 1 |
| TCGA.A3.3387.01 | low | 1 | TCGA.CZ.5465.11 | high | 0 | TCGA.A3.3367.01 | low | 1 |
| TCGA.BP.5168.01 | low | 1 | TCGA.CZ.5468.11 | high | 0 | TCGA.A3.3370.01 | low | 1 |
| TCGA.BP.5169.01 | low | 1 | TCGA.CJ.5689.11 | high | 0 | TCGA.A3.3387.01 | low | 1 |
| TCGA.BP.5180.01 | low | 1 | TCGA.CZ.5469.11 | high | 0 | TCGA.A3.A6NI.01 | low | 1 |
| TCGA.BP.5181.01 | low | 1 | TCGA.B2.5636.01 | high | 1 | TCGA.A3.A6NJ.01 | low | 1 |
| TCGA.BP.5183.01 | low | 1 | TCGA.B2.5641.11 | high | 0 | TCGA.A3.A6NL.01 | low | 1 |
| TCGA.BP.5185.01 | low | 1 | TCGA.B8.4619.01 | high | 1 | TCGA.A3.A8OV.01 | low | 1 |
| TCGA.BP.5190.01 | low | 1 | TCGA.B8.5546.01 | high | 1 | TCGA.AK.3445.01 | low | 1 |
| TCGA.BP.5191.01 | low | 1 | TCGA.B8.5549.11 | high | 0 | TCGA.AK.3455.01 | low | 1 |
| TCGA.BP.5192.01 | low | 1 | TCGA.B8.5552.11 | high | 0 | TCGA.AK.3450.01 | low | 1 |
| TCGA.BP.5196.01 | low | 1 | TCGA.CZ.5456.11 | high | 0 | TCGA.AK.3453.01 | low | 1 |
| TCGA.BP.5199.01 | low | 1 | TCGA.CZ.5457.11 | high | 0 | TCGA.AK.3458.01 | low | 1 |
| TCGA.BP.5201.01 | low | 1 | TCGA.CZ.5455.11 | high | 0 | TCGA.AK.3460.01 | low | 1 |
| TCGA.CJ.4637.01 | low | 1 | TCGA.CZ.5454.11 | high | 0 | TCGA.B0.4701.01 | low | 1 |
| TCGA.CJ.4638.01 | low | 1 | TCGA.CZ.5453.11 | high | 0 | TCGA.B0.4691.01 | low | 1 |
| TCGA.BP.4176.01 | low | 1 | TCGA.CZ.5452.11 | high | 0 | TCGA.B0.4693.01 | low | 1 |
| TCGA.CJ.4876.01 | low | 1 | TCGA.CZ.5461.11 | high | 0 | TCGA.B0.4706.01 | low | 1 |
| TCGA.CJ.4878.01 | low | 1 | TCGA.CZ.5458.11 | high | 0 | TCGA.B0.4707.01 | low | 1 |
| TCGA.CJ.4881.01 | low | 1 | TCGA.CZ.5462.11 | high | 0 | TCGA.B0.4710.01 | low | 1 |
| TCGA.CJ.4885.01 | low | 1 | TCGA.CZ.5463.11 | high | 0 | TCGA.B0.4824.01 | low | 1 |
| TCGA.CJ.4887.01 | low | 1 | TCGA.B8.A54E.01 | high | 1 | TCGA.B0.4713.01 | low | 1 |
| TCGA.CJ.4891.01 | low | 1 | TCGA.BP.4760.01 | high | 1 | TCGA.B0.4714.01 | low | 1 |
| TCGA.BP.4161.01 | low | 1 | TCGA.BP.4334.01 | high | 1 | TCGA.B0.4718.01 | low | 1 |
| TCGA.CJ.4912.01 | low | 1 | TCGA.CZ.4863.11 | high | 0 | TCGA.B0.4811.01 | low | 1 |
| TCGA.CJ.4900.01 | low | 1 | TCGA.CZ.4865.11 | high | 0 | TCGA.B0.4813.01 | low | 1 |
| TCGA.CJ.4901.01 | low | 1 | TCGA.CZ.5451.11 | high | 0 | TCGA.B0.4814.01 | low | 1 |
| TCGA.CJ.4903.01 | low | 1 | TCGA.BP.4784.01 | high | 1 | TCGA.B0.4815.01 | low | 1 |
| TCGA.CJ.5675.01 | low | 1 | TCGA.BP.4769.01 | high | 1 | TCGA.B0.4817.01 | low | 1 |
| TCGA.CJ.4920.01 | low | 1 | TCGA.BP.4795.01 | high | 1 | TCGA.B0.4818.01 | low | 1 |
| TCGA.CJ.4916.01 | low | 1 | TCGA.BP.4994.01 | high | 1 | TCGA.B0.4821.01 | low | 1 |
| TCGA.CJ.5678.01 | low | 1 | TCGA.BP.4995.01 | high | 1 | TCGA.B0.4828.01 | low | 1 |
| TCGA.CJ.5684.01 | low | 1 | TCGA.CJ.5689.11 | high | 0 | TCGA.B0.4833.01 | low | 1 |
| TCGA.CJ.5686.01 | low | 1 | TCGA.CJ.6030.11 | high | 0 | TCGA.B0.4836.01 | low | 1 |
| TCGA.CJ.6027.01 | low | 1 | TCGA.CW.5580.11 | high | 0 | TCGA.B0.4838.01 | low | 1 |
| TCGA.CJ.6028.01 | low | 1 | TCGA.CW.5587.11 | high | 0 | TCGA.B0.5077.01 | low | 1 |
| TCGA.CW.5585.01 | low | 1 | TCGA.CW.6087.11 | high | 0 | TCGA.B0.4844.01 | low | 1 |
| TCGA.CJ.6032.01 | low | 1 | TCGA.CW.6088.11 | high | 0 | TCGA.B0.4845.01 | low | 1 |
| TCGA.CW.5591.11 | low | 0 | TCGA.CW.6090.11 | high | 0 | TCGA.B0.4846.01 | low | 1 |
| TCGA.CZ.4854.01 | low | 1 | TCGA.CJ.5672.11 | high | 0 | TCGA.B0.4847.01 | low | 1 |
| TCGA.B2.5639.01 | low | 1 | TCGA.CJ.5679.11 | high | 0 | TCGA.B0.4849.01 | low | 1 |
| TCGA ID | zDHHC3 | Tumor | TCGA ID | zDHHC3 | Tumor | TCGA ID | zDHHC3 | Tumor |
| TCGA.B2.4101.01 | low | 1 | TCGA.CJ.5681.01 | high | 1 | TCGA.B0.4852.01 | low | 1 |
| TCGA.B4.5377.01 | low | 1 | TCGA.CJ.5681.11 | high | 0 | TCGA.B0.5085.01 | low | 1 |
| TCGA.B0.5092.01 | low | 1 | TCGA.B0.5094.01 | low | 1 |  |  |  |
